# Evaluation of Nitrite, Ethyl Carbamate, and Biogenic Amines in Four Types of Fermented Vegetables

**DOI:** 10.3390/foods10123150

**Published:** 2021-12-20

**Authors:** Yangyang Yu, Yuanshan Yu, Zhenlin Xu

**Affiliations:** 1 Guangdong Provincial Key Laboratory of Food Quality and Safety, College of Food Science, South China Agricultural University, Guangzhou 510642, China; hnyuyang@163.com; 2 Sericultural & Agri-Food Research Institute, Guangdong Academy of Agricultural Sciences/Key Laboratory of Functional Foods, Ministry of Agriculture/Guangdong Key Laboratory of Agricultural Products Processing, Guangzhou 510610, China

**Keywords:** fermented vegetables, nitrite, ethyl carbamate, biogenic amines, detection methods, principal component analysis

## Abstract

Nitrite, ethyl carbamate, and biogenic amines in fermented vegetables are considered harmful compounds. In this study, the concentration of the nitrite, ethyl carbamate, and biogenic amines in four different varieties of fermented vegetables in China was determined. The results show that the nitrite concentration in the fermented cabbage was the highest, followed by fermented mustard, fermented bamboo, and fermented radish. Additionally, nitrite concentration in two fermented cabbage samples and one fermented mustard sample exceeded the maximum allowed residue limit (20 mg/kg) suggested by China’s National Food Safety Standards. However, only one fermented cabbage sample had a very low level of ethyl carbamate (<10 μg/kg). Otherwise, higher biogenic amines were found in the samples of fermented cabbage, fermented bamboo, and fermented mustard. Additionally, the concentration of biogenic amines in some samples exceeded the recommended limit. On the contrary, biogenic amines in fermented radish samples were relatively low. Therefore, the concentration of nitrite and biogenic amine should be closely monitored and controlled during the vegetable fermentation processes, especially for the fermentation processes of bamboo, cabbage, and mustard.

## 1. Introduction

Fermentation is a traditional way of preserving vegetables in China. Various vegetables, including celery, mustard, and potherb [1] can be fomented. The fermented vegetables are prepared as follows: Fresh vegetables are washed with clean water and then sealed in earthenware jars with a 3–6% NaCl solution for 30–40 days at 20–25 °C for fermentation [2]. Despite the health benefits of fermented vegetables, such as decreasing the cholesterol level, promoting digestion, and improving gut health [3,4], nitrite, ethyl carbamate, and biogenic amines present in fermented vegetables can cause health problems.

Nitrite, a potentially cancer-causing compound, is commonly present in fermented vegetables [5]. Hou et al. [6] found that the concentration of nitrite ranges from 0.01 mg/kg to 42.03 mg/kg in fermented vegetables in northeastern China. Liu et al. [7] reported that the nitrite concentration of 65 samples was detected to be above 20 mg/kg among 378 fermented vegetable samples, which is the maximum nitrite concentration in fermented vegetables according to Chinese National Food Standard regulation.

Ethyl carbamate, a probable carcinogen, is widely found in fermented foods and beverages, such as bread, spirits, wines, sake, and soy sauce [8]. During the fermentation process, the chemical reactions of the precursors such as ethanol, urea, and hydrocyanic acid can produce ethyl carbamate [9]. Kim et al. [10] reported that the concentration of ethyl carbamate in Korean-style fermented vegetable, kimchi, ranged from 0 to 16 μg/kg, with a mean value of 4 μg/kg. However, to the best of our knowledge, the studies and surveys on the ethyl carbonate concentration in the Chinese-style fermented vegetable are few.

Biogenic amines are a group of organic nitrogen compounds with low molecular weight. Most of them are produced via enzymatic decarboxylation of amino acids by microorganisms during the fermentation process [11]. Typical biogenic amines are tryptamine, β-phenethylamine, putrescine, cadaverine, histamine, and tyramine [12]. A low dose of biogenic amines can be quickly metabolized in the intestinal tract [13], while a high level of biogenic amines may lead to adverse symptoms, including skin rashes, headache, hot flushes, and nausea [14]. Lee et al. [15] reported that the histamine and tyramine concentrations in fermented onion in Korea exceeded the recommended limits, which posed a risk to the health of consumers.

Fermented bamboo, fermented cabbage, fermented mustard, and fermented radish are four popular fermented vegetables in China. Currently, there is little information on the levels of nitrite, ethyl carbamate, and biogenic amines in the four types of fermented vegetables, which raises concerns regarding the potential health risk associated with consuming these fermented vegetables. Therefore, the objective of this research is to investigate the concentration of nitrite, ethyl carbamate, and biogenic amines in fermented vegetable products. The results provide valuable information to food producers, health professionals, and consumers for developing and consuming healthier fermented vegetables.

## 2. Materials and Methods

### 2.1. Samples

Samples of fermented bamboo, fermented cabbage, fermented mustard, and fermented radish were all purchased on Alibaba.com (accessed on 16 October 2020) (Alibaba Network Technologies Co., Ltd., Guangzhou, China). For each type of fermented vegetable, the top 15 commodities from different brands were selected as the samples for our analysis, according to the sales ranking of fermented mustard on Alibaba.com. Before analysis, the samples were stored at −20 °C. Different brands of fermented bamboo, fermented cabbage, fermented mustard, and fermented radish were labeled FB1–FB15, FC1–FC15, FM1–FM15, and FR1–FR15, respectively. All reagents used for the analyses were of analytical grade reagents and were purchased from Sigma-Aldrich.

### 2.2. Determination of pH Value, Titratable Acidity, and Salinity

pH value was measured using a PHS-3G pH meter (INESA Scientific Instrument Co., Ltd., Hangzhou, China). Titratable acidity (TA) was measured by titrating the brine of each sample with a 0.10 M NaOH solution until the pH reached 8.2 ± 0.2, and the results were expressed as the concentration of lactic acid according to the method described by Liu et al. [16]. Salinity was measured using Mohr’s titration [17], and the detailed process is described as follows: A total of 1 g fermented vegetable sample was combined with 9 mL of distilled water and then homogenized and filtered; Finally, 10 mL of the filtered sample was mixed with 1 mL of 2% potassium chromate indicator and titrated with 0.02 M AgNO_3_ solution until the solution became reddish brown (the endpoint of the titration).

### 2.3. Determination of Nitrite Concentration

The nitrite concentration of each sample was measured using the Griess reaction [18]. Briefly, 4 g of sample was first homogenized with 30 mL of distilled water. Then, the homogenized sample was heated at 95–100 °C for 20 min and immediately cooled in an ice bath. Next, the sample was combined with 0.5 mL of 30% *w*/*v* ZnSO_4_·7H_2_O solution and 0.5 mL of 15% (*w*/*v*) K_4_Fe (CN)_6_·3H_2_O solution, followed by shaking. After filtration, the sample solution was sequentially mixed with 1 mL of 0.2% sulfanilamide, and 1 mL of 0.1% *N*-1-naphtyethylene diamine dihydrochloride, which are color development reagents. This color development reaction was carried out at room temperature for 15 min. The optical density (OD) of each sample was obtained at a wavelength of 538 nm with a reagent blank as a reference. The standard curve was obtained by performing the same color development process and OD analysis.

### 2.4. Determination of Ethyl Carbamate Concentration

The ethyl carbamate concentration in each sample was measured using a gas chromatography–mass spectrometry (GC–MS) method [19]. For each analysis, 4 g of sample was homogenized with 4 mL of distilled water and then sonicated for 10 min. After that, the mixture was kept static in a solid-phase extraction column (ANPEL Laboratory Technologies Inc., Shanghai, China) for 10 min. Next, the mixture was washed with hexane and eluted with 10 mL of ethyl acetate–diethyl ether solution (5:95, *v*/*v*). Then, the eluent was evaporated to 0.5 mL via N_2_ flow at room temperature and brought to a volume of exactly 1 mL for testing.

The GC–MS analysis was conducted with an Agilent 7890A-5975C equipped with a DB-WAX column (30 m × 0.25 mm I.D., 0.25 μm df; Agilent Technologies, Inc., Santa Clara, CA, USA). Helium was used as the carrier gas and the flow rate was 1.0 mL/min. Sample aliquots (2.0 µL/sample) were injected under splitless mode. The temperature of the GC oven was programmed as follows: holding at 50 °C for 1 min, ramping to 180 °C at a rate of 8 °C/min, and ramping to 240 °C at a rate of 40 °C/min, followed by holding at 240 °C for 5 min. The mass spectral analysis was performed in a 70 eV electron impact ionization mode; the temperatures of the injector port and mass spectrometer interface lines were 220 °C and 250 °C, respectively. For qualitative analysis, the mass spectrometer was conducted in scan mode, while for quantitative analysis, the mass spectrometer was conducted under the selected ion monitoring (SIM) mode. The ethyl carbamate fragment ions have m/z values of 44, 62, 74, and 89. The calibration curves of ethyl carbamate were constructed using peak area.

### 2.5. Determination of Biogenic Amine Concentrations

The concentrations of biogenic amines were analyzed according to the method developed by Zhang et al. [20] with slight modifications: First, 1 g of a sample was homogenized with 3 mL of 0.4 M HClO_4_ and extracted with ultrasound (80 W, 50 °C, 1 h). Then, the mixture was centrifuged at 10,000× *g* for 5 min, and the supernatant was collected. Next, 250 μL of the supernatant was combined with 25 μL of NaOH solution (2 M), 75 μL of saturated NaHCO_3_, and 500 μL of dansyl chloride (5 mg/mL). The mixture was heated at 55 °C for 40 min. Next, 25 μL of NH_4_OH solution (25%) was added to the mixture, which was incubated at 55 °C for another 10 min. After being filtered by a membrane with a pore size of 0.22 μm, the treated sample was analyzed by an Agilent high-performance liquid chromatography system. The system was equipped with an Eclipse XDB-C18 column (dimensions: 4.6 mm × 250 mm, particle size: 5 µm) and a 254 nm UV detector. The separation of the amines was achieved using a linear gradient of acetonitrile/H_2_O mixtures as the mobile phase. The gradient elution program was as follows: 0–4 min, 50% acetonitrile; 4–22 min, 50–90% acetonitrile; 22–30 min, 90–50% acetonitrile; 30–35 min, 50% acetonitrile. The injection volume, flow rate, and column temperature were 10 µL, 0.8 mL/min, and 25 °C, respectively.

### 2.6. Method Validation

The method validation was based on accuracy, precision, and sensitivity. The calibration curves and the related linearities (coefficients of determination, R^2^) were obtained using the linear regression method. Accuracy refers to how close a measurement is to the theoretical value, while precision refers to how close the measurements of the same sample are to each other. Matrix spikes with a nitrite concentration of 50, 20, or 5 mg/kg, an ethyl carbamate concentration of 20, 10, or 5 μg/kg, and a biogenic amine concentration of 100, 50, or 20 mg/kg were employed in the analysis. The accuracy was evaluated based on the recovery rate (RR) of the spiked sample analysis. The intra- and inter-day percent relative standard deviations (RSD %) were determined. The limit of detection (LOD) and limit of quantification (LOQ) were used to evaluate the sensitivity of the methods. LOD is defined as the concentration of an analyte with a signal-to-noise (S/N) ratio of 3:1, while LOQ is defined as the concentration of an analyte with an S/N ratio of 10:1 [21].

### 2.7. Statistical Analysis

All tests were carried out in triplicate. All data in this manuscript are expressed as mean ± standard deviation (SD). Analysis of variance (ANOVA) testing was conducted using SPSS17.0 (SPSS, Inc., Chicago, IL, USA), and Duncan’s test (*p* < 0.05) was adopted for the comparisons between multiple means. The plots were plotted using Origin v2021b (Origin Lab Corporation, Northampton, MA, USA).

## 3. Results and Discussion

### 3.1. Method Validation

The methods for nitrite, ethyl carbamate, and biogenic amine analyses were validated, and the obtained statistical data are listed in Table 1. The accuracies and precisions of the analysis methods can be assessed based on the RR values and the RSD values, respectively. For the majority of analyses, the RR values rangeed within 81−124%, and the RSD values were below 11%. As suggested by the FDA guidance document [22], precision below 16% is acceptable for bioanalytical method validation, suggesting the analytical methods used in this study were reliable. The sensitivities of the analysis method can be found based on the LOD and LOQ values, respectively [21]. The LOD and LOQ values of the nitrite analysis were 0.22 mg/kg and 0.74 mg/kg, respectively; the LOD and LOQ values of the ethyl carbamate analysis were 2.12 μg/kg and 7.07 μg/kg, respectively; the LOD and LOQ values of biogenic amine analyses ranged from 0.16 mg/kg to 0.39 mg/kg and from 0.53 mg/kg to 1.31 mg/kg, respectively. Moreover, all these parameters were well below the maximum residue limits for fermented vegetables. These results suggested that those analysis methods had high sensitivity and were suitable for analyzing nitrite, ethyl carbamate, and biogenic amines.

### 3.2. pH, Titratable Acidity, and Salinity

**Figure 1:**
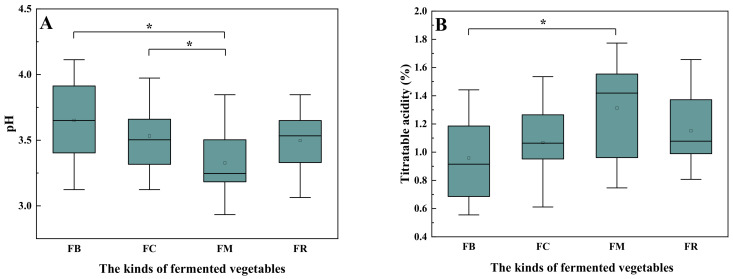

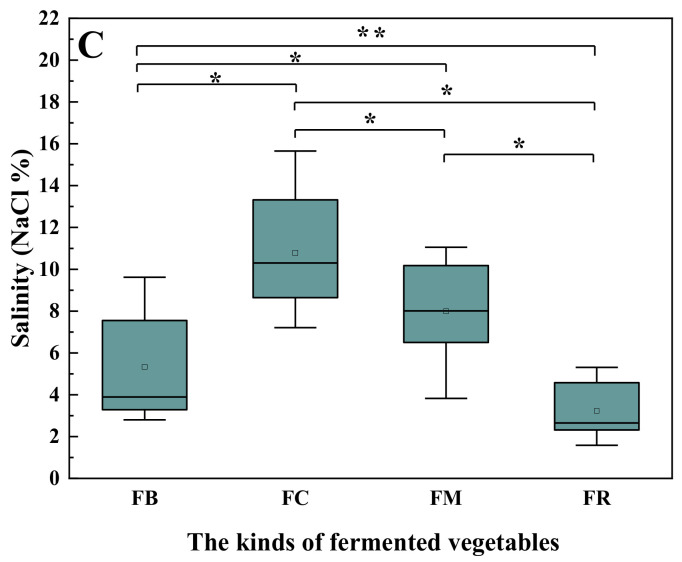
Physicochemical characteristics of different types of fermented vegetables: (**A**) pH, (**B**) titratable acidity, and (**C**) salinity. FB, fermented bamboo; FC, fermented cabbage; FM, fermented mustard; FR, fermented radish; * indicates significantly different means at *p* < 0.05; ** indicates significantly different means at *p* < 0.01.

The characteristics and flavor of fermented vegetables are associated with pH, titratable acidity, and salinity [23]. Overall, the pH of the fermented mustard samples was the highest among the four types of fermented vegetables, which was significantly lower than the samples of fermented cabbage and fermented radish. The titratable acidity of the fermented mustard samples was the highest among the four types of fermented vegetables, which was significantly higher than the samples of fermented bamboo, shown in Figure 1B. Generally, fermented vegetables are considered to be ripe when the pH is below 4.0, and the TAA is greater than 0.3% [24]. In this study, a pH over 4.0 was only found in one sample of fermented bamboo, and the TAA was over 0.3% in all samples. This result was consistent with previous reports on fermented vegetables. The salinity values of the four types of fermented vegetables showed large differences (Figure 1C). The average salinity of fermented cabbage samples was 10.78%, which was the highest among the four types of fermented vegetables. The average salinity of fermented radish samples was the lowest. The differences in salinity were indicative of consumer preference regarding different fermented vegetables, as fermented vegetables are often cooked with other vegetables, and consumers have different taste preferences.

### 3.3. Nitrite

The nitrite concentration data of the four types of fermented vegetables are shown in Table 2. The average nitrite concentrations in fermented bamboo, fermented cabbage, fermented mustard, and fermented radish samples were 2.93 ± 2.04 mg/kg, 12.13 ± 7.76 mg/kg, 7.46 ± 5.54 mg/kg, and 1.82 ± 2.05 mg/kg, respectively. The average nitrite concentration in fermented radish samples was the lowest among the four types of fermented vegetables, while the average nitrite concentration in fermented cabbage samples was the highest. Ding et al. [5] reported that the concentration of nitrite in kimchi was 14 mg/kg, which is much higher than those found in red cabbage and fermented olives (2.0 mg/kg and 3.0 mg/kg, respectively). These results agreed with our results, suggesting that the difference in nitrite concentrations among different types of fermented vegetables was large. As the nitrite in fermented vegetables is reduced from nitrate during the fermentation process, the large difference in nitrite concentrations among different types of vegetables may be due to the significant difference in nitrite concentrations among different types of fresh vegetables.

**Figure 2:**
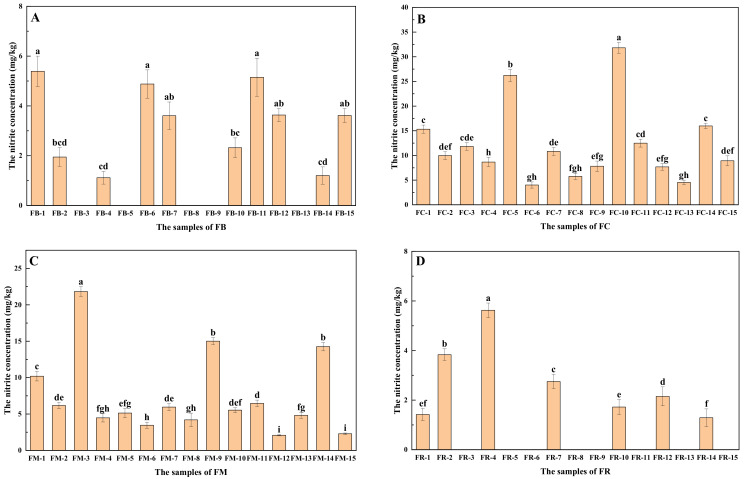
The concentration of nitrite in different types of fermented vegetables: (**A**) fermented bamboo, (**B**) fermented cabbage, (**C**) fermented mustard, and (**D**) fermented radish. FB, fermented bamboo; FC, fermented cabbage; FM, fermented mustard; FR, fermented radish. The different letters (a, b, c, etc.) indicate significantly different (Duncan’s test, *p* < 0.05).

As shown in Figure 2, the nitrite concentration varied significantly between the same type of fermented vegetable samples. This is because the nitrate content is also affected by fermentation temperature, fermentation time, and NaCl concentration. In China, the maximum allowed nitrite content in fermented vegetables is 20 mg/kg. In this study, the nitrite concentration in two fermented cabbage samples (13.33% of the total fermented cabbage samples) and one fermented mustard sample (6.67% of the total fermented mustard samples) were above 20 mg/kg (Figure 2B,C). The literature on the nitrite concentration in fermented vegetables is scarce. Among fermented vegetables, fermented cabbage is the most widely studied. Additionally, Liu et al. reported that the nitrite concentrations of 118 fermented cabbage samples were high, ranging from 5.8 mg/kg to 32.4 mg/kg, with an average of 17.2 ± 2.1 mg/kg [7]. Among the 118 samples, 24 samples had a nitrite concentration above 20 mg/kg, which was 20.34% of the total samples. These results suggested that the high nitrite levels in fermented cabbage and fermented mustard pose health risks to consumers, and controlling nitrite concentration in fermented vegetables is urgently needed.

### 3.4. Ethyl Carbamate

In this study, one fermented cabbage sample showed a low level of ethyl carbamate (<10 μg/kg), and other fermented vegetable samples had no detectable ethyl carbamate. Similarly, Hasnip et al. [25] studied the concentration of ethyl carbamate in 23 pickled vegetable samples and found that only one sample had a detectable ethyl carbamate of 8 µg/kg. At present, regulations regarding ethyl carbamate concentration have only been implemented on alcoholic beverages. For example, in Canada, the maximum allowed ethyl carbamate levels in alcoholic beverages are 30 µg/kg, 100 µg/kg, 150 µg/kg, 200 µg/kg, and 400 µg/kg for wine, fortified wine, distilled spirits, sake, and fruit brandies, respectively [26]. Therefore, the results of this study showed that the chance of excessive ethyl carbamate intake associated with consuming fermented vegetables is very low.

### 3.5. Biogenic Amine

As shown in Table 3, among the four types of fermented vegetables, fermented cabbages had the highest average concentrations of putrescine (54.75–319.41 mg/kg in range and 153.53 ± 82.38 mg/kg on average), cadaverine (13.03–262.46 mg/kg in range and 105.26 ± 67.98 mg/kg on average), histamine (2.29–70.17 mg/kg in range and 45.44 ± 19.16 mg/kg on average), tryptamine (8.98–66.04 mg/kg in range and 20.22 ± 14.71 mg/kg on average), β-Phenethylamine (2.58–26.68 mg/kg in range and 13.42 ± 6.12 mg/kg on average); fermented bamboos had the highest average concentrations of spermidine (2.58–154.84 mg/kg in range and 38.96 ± 42.19 mg/kg on average) and spermine (0.59–23.14 mg/kg in range and 7.92 ± 6.76 mg/kg on average). In addition, the concentration of biogenic amines in fermented radish was relatively low, and spermine was not detected in all fermented radish samples. Statistical analysis of these data showed significant differences in biogenic amine concentration among the four types of fermented vegetables. This is due to the difference in precursors, microorganisms, and conditions for cell proliferation [13]. In addition, the biogenic amines of the four types of fermented vegetables mainly consisted of putrescine, cadaverine, tyramine, and histamine, and the concentration of β-phenethylamine and spermine were relatively low in all four types of fermented vegetables. Similarly, Mayr and Schieberle [27] investigated the biogenic amine concentration in sauerkraut and observed that putrescine (108.9 mg/kg on average), tyramine (60.66 mg/kg on average), and histamine (37.01 mg/kg on average) were the main biogenic amine species, followed by cadaverine (21.5 mg/kg on average) and spermidine (10.98 mg/kg on average). The authors also found that the concentrations of spermine (1.2 mg/kg on average) and β-phenethylamine (0.73 mg/kg on average) were very low.

**Figure 3:**
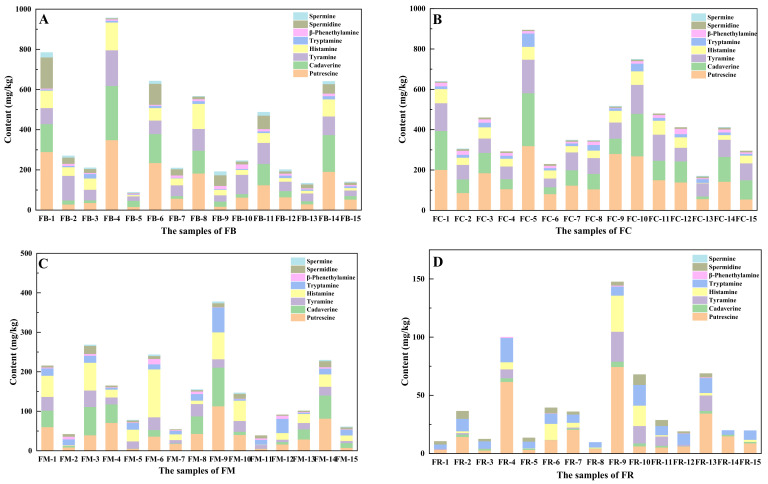
The concentration of biogenic amines in different types of fermented vegetables: (**A**) fermented bamboo, (**B**) fermented cabbage, (**C**) fermented mustard, and (**D**) fermented radish. FB, fermented bamboo; FC, fermented cabbage; FM, fermented mustard; FR, fermented radish.

Excess intake of biogenic amines can lead to various health problems. At present, there are no government regulations on the concentration of biogenic amines in fermented vegetables. To the best of our knowledge, histamine is the only biogenic amine that has a government regulation in fish products: 50 mg/kg limit according to the US Food and Drug Administration [22]. Several researchers have proposed concentration limits of biogenic amines in food: 100–800 mg/kg for tyramine, 100 mg/kg for histamine, 30 mg/kg for β-phenylethylamine, and 1000 mg/kg for total biogenic amines [28,29]. As shown in Figure 3A, fermented bamboo samples with a histamine concentration above 100 mg/kg were FB-4 (138.13 ± 16.46 mg/kg) and FB-8 (124.72 ± 20.81 mg/kg); fermented bamboo samples with a tyramine concentration above 100 mg/kg were FB-4 (177.68 ± 11.75 mg/kg), FB-8 (108.31 ± 11.28 mg/kg), and FB-11 (104.88 ± 8.52 mg/kg); the fermented bamboo sample with a β-Phenethylamine concentration above 30 mg/kg was FB-10 (31.53 ± 3.05 mg/kg). As shown in Figure 3B, there was no fermented cabbage samples with a histamine concentration above 100 mg/kg or a β-phenethylamine concentration above 30 mg/kg; fermented cabbage samples with a tyramine concentration above 100 mg/kg were FB-1 (137.55 ± 9.38 mg/kg), FB-5 (166.14 ± 6.75 mg/kg), FB-10 (143.54 ± 12.97 mg/kg), and FB-11 (129.21 ± 9.07 mg/kg). As shown in Figure 3C, the fermented mustard sample with a histamine content above 100 mg/kg was FM-6 (121.30 ± 1.75 mg/kg). As shown in Figure 3D, the contents of histamine, tyramine, and amphetamine in fermented radish samples were all below their limits. Hence, fermented bamboo had a probability of exceeding the maximum allowed concentrations of histamine, tyramine, and amphetamine; fermented cabbage had a risk of exceeding the maximum allowed concentration of tyramine and amphetamine; fermented mustard had a risk of exceeding the maximum allowed concentration of histamine; however, fermented radish had no risk of exceeding the maximum allowed concentration of biogenic amines.

## 4. Conclusions

The concentration of the nitrite, ethyl carbamate, and biogenic amines in four types of Chinese fermented vegetables (15 samples per type of fermented vegetable) were studied. The nitrite concentration in two fermented cabbage samples and one fermented mustard sample exceeded the maximum allowed residue limit (20 mg/kg) suggested by China’s National Food Safety Standards. However, ethyl carbamate was only found in one fermented cabbage sample, and its concentration was below 10 μg/kg. Hence, the risk of excessive ethyl carbamate intake associated with consuming fermented vegetables was extremely low. In addition, the concentrations of biogenic amines in fermented bamboo, fermented cabbage, and fermented mustard samples were high, and the concentration of biogenic amines in some samples exceeded the recommended limit. Therefore, to minimize the health risk, the concentrations of nitrite and biogenic amines should be closely monitored and controlled during the vegetable fermentation processes, especially for the fermentation processes of bamboos, cabbages, and mustard.

## Figures and Tables

**Table 1:** Statistical data of method validation tests for nitrite, ethyl carbamate, and biogenic amines. RSD: relative standard deviations; LOD: limit of detection;LOQ: limit of quantification.

	Fortification Level	Recovery (%)	RSD (%)	LOD	LOQ
Nitrite	50 mg/kg	81.1 ± 9.7	10.1	0.22	0.74
	20 mg/kg	85.6 ± 13.8	10.8		
	5 mg/kg	108.7 ± 10.7	3.83		
Ethyl Carbamate	20 µg/kg	110 ± 5	4.86	2.12	7.07
	10 µg/kg	91.1 ± 6.2	3.60		
	5 µg/kg	89.4 ± 9.3	3.75		
Putrescine	100 mg/kg	117.6 ± 5.3	1.90	0.25	0.84
	50 mg/kg	115.6 ± 2.4	2.37		
	20 mg/kg	96.0 ± 5.8	0.65		
Cadaverine	100 mg/kg	124 ± 10	3.77	0.22	0.73
	50 mg/kg	102 ± 21	5.46		
	20 mg/kg	94.0 ± 10.7	1.36		
Tyramine	100 mg/kg	123 ± 7	7.01	0.25	0.83
	50 mg/kg	118 ± 6	6.48		
	20 mg/kg	112 ± 2	2.45		
Histamine	100 mg/kg	117 ± 12	5.87	0.28	0.95
	50 mg/kg	117 ± 4	3.67		
	20 mg/kg	108 ± 7	7.05		
Tryptamine	100 mg/kg	107 ± 8	6.76	0.39	1.31
	50 mg/kg	107 ± 7	5.55		
	20 mg/kg	101 ± 6	4.60		
β-Phenethylamine	100 mg/kg	112 ± 10	8.89	0.24	0.79
	50 mg/kg	109 ± 4	3.32		
	20 mg/kg	104 ± 6	4.36		
Spermidine	100 mg/kg	110 ± 18	6.69	0.16	0.53
	50 mg/kg	112 ± 13	3.17		
	20 mg/kg	113 ± 7	0.83		
Spermine	100 mg/kg	115 ± 5	3.41	0.29	0.98
	50 mg/kg	109 ± 6	3.67		
	20 mg/kg	109 ± 16	6.88		

**Table 2:** **Nitrite concentrations in different types of fermented vegetables:** FB, fermented bamboo; FC, fermented cabbage; FM, fermented mustard; FR, fermented radish; LOD: limit of detection. Mean ± SD (standard deviation) values in the same row, followed by different letters (a, b, c, etc.) are significantly different (Duncan’s test, *p* < 0.05).

The Different Kind of Fermented Vegetables		FB	FC	FM	FR
Nitrite concentration (mg/kg)	Mean ± SD	2.93 ± 2.04 c	12.13 ± 7.76 a	7.46 ± 5.54 b	1.82 ± 2.05 c
	Range	<LOD–5.39	1.43–31.82	0.81–22.83	<LOD–5.62
Number of samples (Nitrite content higher than 20 mg/kg)		0	2	1	0

**Table 3:** **The concentrations of biogenic amines in different types of fermented vegetables:** FB, fermented bamboo; FC, fermented cabbage; FM, fermented mustard; FR, fermented radish; LOD: limit of detection. Mean ± SD (standard deviation) values in the same row followed by different letters (a, b, c, etc.) are significantly different (Duncan’s test, *p* < 0.05).

Different Group of Samples		FB	FC	FM	FR
Putrescine	Mean ± SD	116.12 ± 107.79 a	153.53 ± 82.38 a	38.47 ± 31.68 b	18.2 ± 22.07 b
	Range	16.61–349.06	54.75–319.41	5.30–113.21	2.71–61.70
Cadaverine	Mean ± SD	75.96 ± 79.25 a	105.26 ± 67.98 a	29.11 ± 29.99 b	1.53 ± 1.25 b
	Range	12.92–269.73	13.03–262.46	0.26–97.76	<LOD–4.55
Tyramine	Mean ± SD	74.85 ± 42.82 a	91.78 ± 35.18 a	19.53 ± 12.09 b	4.96 ± 7.66 b
	Range	22.15–177.68	43.73–166.14	1.10–41.72	<LOD–25.73
Histamine	Mean ± SD	52.06 ± 40.7 a	45.44 ± 19.16 a	34.93 ± 32.78 a	5.33 ± 8.85 b
	Range	6.52–138.13	2.29–70.17	0.62–70.23	<LOD–17.47
Tryptamine	Mean ± SD	8.95 ± 5.44 b	20.22 ± 14.71 a	17.12 ± 14.9 ab	8.94 ± 4.96 b
	Range	2.71–22.32	8.98–66.04	3.05–62.79	3.45–20.86
β-Phenethylamine	Mean ± SD	9.52 ± 4.97 b	13.42 ± 6.12 a	3.98 ± 3.49 c	0.28 ± 0.29 d
	Range	4.81–22.54	2.58–31.53	0.77–13.53	<LOD–0.57
Spermidine	Mean ± SD	38.96 ± 42.19 a	6.11 ± 1.41 b	6.81 ± 5.48 b	2.73 ± 2.53 b
	Range	2.60–154.84	3.82–8.65	1.10–20.39	<LOD–8.68
Spermine	Mean ± SD	7.92 ± 6.76 a	0.5 ± 0.51 b	0.95 ± 1.16 b	<LOD
	Range	0.59–23.14	<LOD–1.29	<LOD–3.41	<LOD
Total biogenic amines	Mean ± SD	384.32 ± 272.01 a	426.96 ± 202.08 a	151.02 ± 99.02 b	43.06 ± 41.39 b
	Range	88.58–955.91	169.73–893.77	38.36–377.43	9.53–156.43
